# Contextual factors that affect diabetic retinopathy screening uptake at township health units in southern China: a qualitative study with service providers and users

**DOI:** 10.1136/bmjopen-2025-109139

**Published:** 2026-05-12

**Authors:** Baixiang Xiao, Ving Fai Chan, Carlos Price-Sanchez, Mapa Prabhath Piyasena, Yanfang Wang, Qiong Wan, Nathan Congdon

**Affiliations:** 1 Affiliated Eye Hospital, Jiangxi Medical College, Nanchang University, Nanchang, Jiangxi, China; 2 Centre for Public Health, Queen's University Belfast, Belfast, UK; 3 College of Health Sciences, University of KwaZulu-Natal, Durban, South Africa; 4 Vision and Eye Research Institute, Anglia Ruskin University School of Medicine, Chelmsford, UK; 5 The State Key Laboratory of Ophthalmology, Zhongshan Ophthalmic Center, Sun Yat-Sen University, Guangzhou, Guangdong, China; 6 Shaozhou People’s Hospital, Shaoguan City, Guangdong, China; 7 ORBIS International, New York, New York, USA

**Keywords:** China, DIABETES & ENDOCRINOLOGY, Diabetic retinopathy, Health Services

## Abstract

**Abstract:**

**Objectives:**

This study aimed to identify the factors that influence access to diabetic retinopathy screening (DRS).

**Design:**

This is a qualitative case study.

**Setting:**

Township health units in Shaoguan City, Guangdong Province, China.

**Participants:**

This study included two representative patient groups (n=15) and five health-staff groups (n=42).

**Primary and secondary outcome measures:**

Focus group discussions were guided by a female ophthalmologist and other ophthalmology staff to determine the contextual factors influencing DRS uptake in people with diabetes mellitus in Qujiang District, Shaoguan City, southern China. Directly observed treatment and short-course (DOTS) components for the assessment of tuberculosis services were referred to for health structure when themes were extracted using deductive thematic analysis.

**Results:**

By referring to DOTS components related to the government, case detection, treatment, drug supply and recording system, we identified 31 factors associated with DRS uptake. Among these, six were from the perspective of service users whereas the remaining 25 were related to providers. From these factors, 10 modifiable themes pertained to policy, financing, interdepartmental coordination, hospital preparedness, primary healthcare staff training and public awareness through health education and quality enhancement of public health services. Two unmodifiable factors were also extracted: discomfort from pupil dilation during the examination and long travel distance to the facility.

**Conclusions:**

This analysis identified contextual factors influencing DRS uptake, including policy, financing and public awareness, which, if addressed, could significantly enhance future screening uptake and disease management.

**STRENGTH AND LIMITATIONS OF THIS STUDY**

- This was a pragmatic service-level study embedded within an ongoing diabetic retinopathy screening pilot programme in southern China.
- This method reinforces the value of embedded implementation research in adaptive health system reform.
- It focused primarily on healthcare providers, with limited input from unregistered or rural patients due to the convenience of the purposive sampling at the Zhenjiang District People’s Hospital.

## Introduction

 Diabetic retinopathy (DR) is a leading cause of vision loss among people with diabetes mellitus (PwDM), and regular diabetic retinopathy screening (DRS) is a cost-effective strategy for preventing the progression to sight-threatening disease.^1 2^ However, across diverse health systems globally, achieving high DRS uptake remains a persistent challenge.^3^,^5^ Studies revealed that low uptake is associated with a higher risk of sight-threatening diabetic retinopathy,^5^ while consistent participation significantly improves health outcomes and reduces long-term healthcare costs.^3^,^5^

Numerous studies have examined the factors affecting adherence to DRS programmes.^6^,^9^ For example, a systematic review found that social deprivation, rather than geographic distance, was consistently associated with poor screening attendance.^3^ Qualitative studies across countries have revealed critical contextual enablers and barriers.^6^,^810^ In the UK, strong collaboration between primary care providers and the integration of DRS into routine services have been emphasised as key strategies.^11^ A study in Sri Lanka highlighted the importance of improving awareness and accessibility, highlighting how both provider-side delivery and user-side engagement shape screening uptake.^6^ A systematic review of attitude toward telemedicine-based retinopathy screening detected the importance of addressing patients’ knowledge gaps, financial barriers and provider training, alongside streamlined referral systems, could enhance screening uptake and effectiveness.^9^ These findings underscore the importance of understanding how health systems and community dynamics interact in specific settings.

In China, people with diabetes are encouraged to register in both primary care and higher levels of the healthcare system.^12^ Registration at secondary and tertiary hospitals, especially those with endocrinology units, allows for streamlined chronic disease management and benefits, such as waived consultation fees and centralised medical records. Many institutions deliver free health talks as part of their patient education strategies.^9^ However, little is known about how these systemic features affect participation in DRS, particularly in township health units (THUs) that serve peri-urban and rural populations.

In 2017, a DR screening initiative was launched at Zhenjiang District People’s Hospital (ZPH, now known as Shaozhou People’s Hospital) in Shaoguan City, Guangdong Province, southern China.^13 14^ Two free screening delivery models were implemented: (1) a mobile DRS team that visited THUs and (2) hospital-based screening through outpatient services. Eye examination for the DRS included vision acuity, slit lamp, intraocular pressure and fundus images taken with camera Canon CR-2 when pupil dilated.^12^ Despite these efforts, uptake remained low during the early stages, ranging from 20% to 50% across towns, far below the uptake levels seen in the UK, where structured DRS programmes achieved rates of 50–80% in the first few years.^13^

This study aimed to explore the contextual factors, both health system-related and sociocultural, affecting the delivery and uptake of DRS in Shaoguan, as perceived by both healthcare providers and service users. By examining experiences at the township level, this qualitative research sought to generate insights that can inform strategies to improve screening participation in similar settings across China.

## Methods

This qualitative case study was part of a larger DRS study with some findings published,^12 14^ and ethical approval was obtained from the medical ethics committee of Zhongshan Ophthalmic Center, Sun Yat-sen University (approval no. 2019KYPJ067) as part of the wider study. Written informed consent was obtained from all participants, including patient participants (PPs) and village doctors (VDs) from different counties in Shaoguan City, staff from the ZPH and local health officers. This study adhered to the ethical principles outlined in the Declaration of Helsinki.

### Participant selection and recruitment

The participants were purposively sampled to ensure diversity across key demographics and professional roles. The sample included PwDM registered at ZPH (both attendees and non-attendees of DRS), with variation in location (urban vs rural), gender and age. Healthcare professionals involved in diabetes or eye care included trained DRS team members and public health officers from ZPH, the Bureau of Health (BOH), public health staff from THUs and VDs from Shaoguan. Because the study aimed to identify service delivery barriers, the majority of focus groups involved healthcare providers responsible for delivering DRS services. All participants were informed of the researchers and the purpose of the study at the invitation.

In total, seven groups (patients: two groups, service supplier: five groups, table 1) participated in structured group discussions, each lasting approximately 60 min. These groups were selected to provide a broad range of perspectives from patients and healthcare workers involved in DRS delivery and utilisation. The sample size and selection of participants were agreed on by the survey team, hospital leaders and BOH for the consideration of data saturation. All the invited healthcare professionals participated, while one patient in the first PP group missed the appointment due to family responsibilities. All group discussions were arranged within 1 week of early 2019.

**Table 1:** Number of group discussions conducted for the qualitative study in Shaoguan in 2019

Item	Name of the groups	No. of participants
1	Patient group 1	7
2	Patient group 2	8
3	Screening team (three ophthalmologists ever been to the screening, three nurses, one technician and one administration)	8
4	Health officers from the prefecture (three), district BOH (four) and ZPH (two)	9
5	General practitioners or public health doctors from THUs	10
6	Village doctor group 1	8
7	Village doctor group 2	7
	**Total**	**57**

BOH, Bureau of Health; THU, township health unit; ZPH, Zhenjiang District People’s Hospital.

### Patients and public involvement

All participants, including patients, were informed of the study questions before the group discussions. PPs were not involved in this report nor are the dissemination plans for our research.

### Data collection

Group discussions were held in a quiet, separate conference room at ZPH and were observed by the head nurse of the eye department. They were facilitated by an experienced female ophthalmologist and project director (BX), who designed the entire programme and the incorporated studies, and was familiar with the interventions, the research setting and the project personnel involved at ZPH. Through the programme, she knew the local BOH staff and some VDs rather than any PPs. The head nurse knew most of the PPs through the DM registration system and routine DM management at the ZPH.

The discussions were guided by two core questions developed by experienced eye care programme staff in consultation with local health authorities. These were based purely on programme experiences, not on any theory or literature review.

- What challenges do PwDM face in accessing diabetes care?
- What are the reasons for non-attendance at DRS?

These two questions were pre-tested among the three project-staff and two patients from the communities. The participants were encouraged to explore both modifiable and non-modifiable factors, with particular attention paid to the former. Neutrality was maintained through the use of open-ended questions, encouragement of contrasting views and reflexive practices. The directly observed treatment, short-course (DOTS) framework^15^ originally developed for tuberculosis control was adapted as a conceptual model for structuring the discussion until no more factors could be identified and data saturation was reached in the opinion of the investigators. The DOTS framework includes five components: government commitment, case detection, standardised treatment, uninterrupted supply of resources and standardised reporting.

Data collection continued until thematic saturation was reached. After each focus group discussion, transcripts and field notes were reviewed by the research team and preliminary factors influencing DRS uptake were identified. As additional discussions were analysed, newly identified factors were compared with existing codes. When subsequent discussions produced no new factors and the existing themes were consistently repeated across participant groups, the research team concluded that thematic saturation had been achieved through team consensus.

All discussions were audio-recorded in Mandarin Chinese and/or the local dialect and supplemented by handwritten notes from two team members (YW and QW). At the end of each session, the elicited factors were summarised and fed back to participants for confirmation. The transcripts were prepared in Chinese, translated into English and double-checked by YW and BX.

### Data analysis

Deductive thematic analysis was conducted using the five components of the DOTS framework as predefined analytical domains: government commitment, case detection, standardised treatment, uninterrupted supply of resources and standardised reporting systems. BX and YW manually coded the transcripts, which were double-checked with voice records, and the themes were reviewed and validated by a senior researcher (VFC).

**Figure 1:**
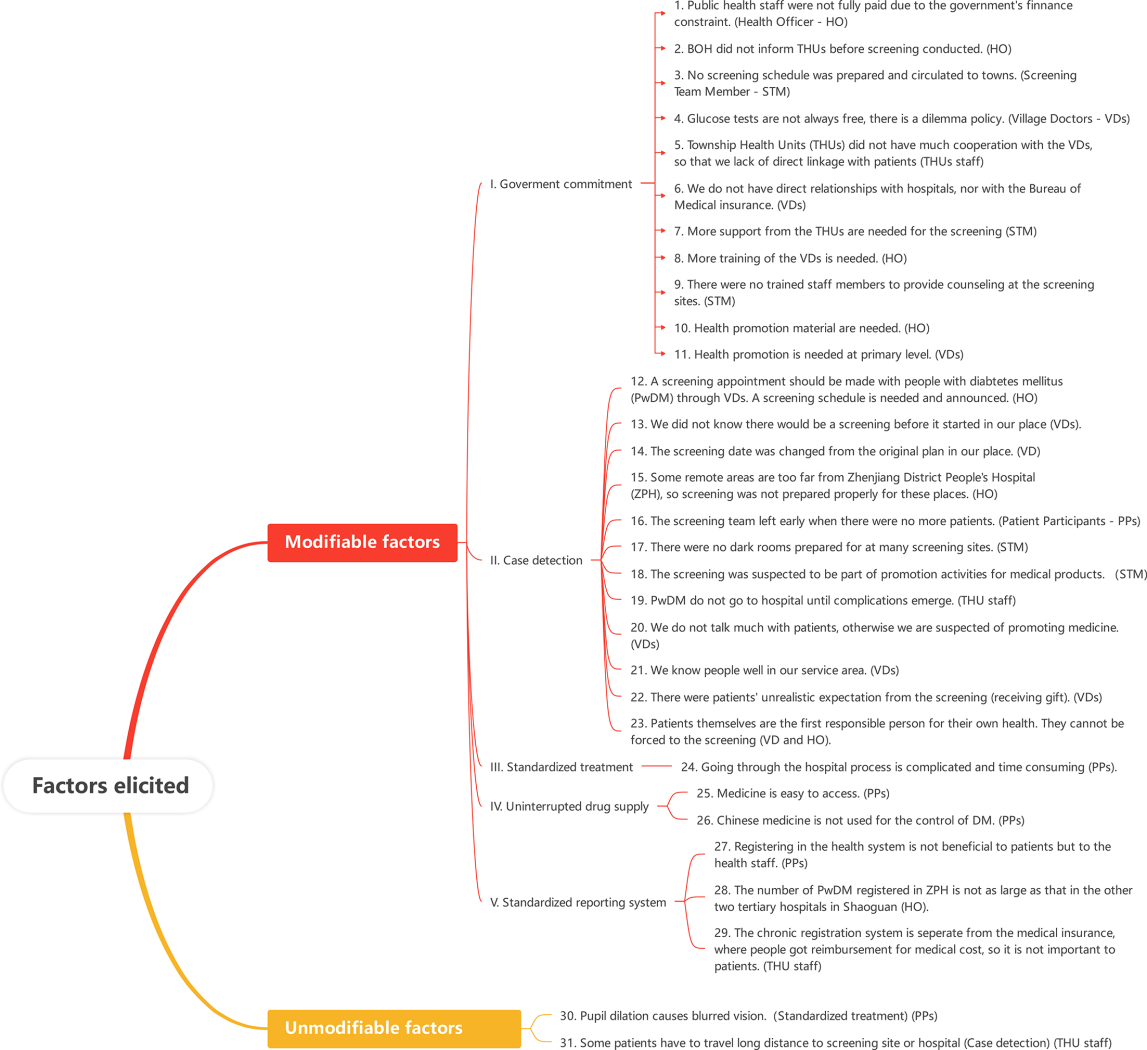
List of the 31 factors elicited from the qualitative interviews in Shaoguan, China in 2019. BOH, Bureau of Health.

Each factor was then mapped to the DOTS component that best represented the health system function in which it occurred. Where factors could reasonably relate to more than one component (for example, health education influencing both community awareness and case detection), categorisation was based on the factor’s primary operational role within the screening programme. Discrepancies in coding were discussed among the research team until consensus was reached. A thematic tree linking the identified factors to the corresponding DOTS components has been added to enhance transparency of the analytical process (figure 1).

The team reported the study in accordance with the 32-item Consolidated Criteria for Reporting Qualitative Research (COREQ) checklist.

## Results

The study included 15 PPs (acceptance rate 15/16=93.8%), aged 39–73 years (table 2) in two groups. Among them, four lived in rural areas, nine were female, 14 had type II DM and one had type I DM. Almost all PPs (14/15=93.3%) reported managing their glucose levels with medications, including insulin. Most (32/42=76.2%) of the healthcare provider participants were between 35 years and 45 years of age, with some exceptions; one ophthalmologist was aged 58 years, two BOH staff members were over 55 years and seven general practitioners and VDs were over 55 years (table 3).

**Table 2:** The basic information of the patient participants in the group discussions in Shaoguan, 2019

Participants’ basic information	Group 1	Group 2
Number of participants	7	8
Average age (years)	62.2	53.0
Age range (years)	46–73	39–73
No. of females in the group	5	4
Type of DM: T1DM	1	0
T2DM or unclear	6	8
Range of DM duration (years)	4–22	1–8
Number of participants diagnosed with DR	0	0
Number of participants with visual impairment	1	0

DM, diabetes mellitus; DR, Diabetic Retinopathy; T1DM, type I diabetes mellitus; T2DM, type 2 diabetes mellitus.

**Table 3:** The basic characteristics of service-supplier participants in the group discussion in Shaoguan, 2019

Name of the group	No. of participants	% among service providers	Age range, years	Average age, years	Years at their posts, years
Screening team	8 (five female)	19.05	35–58	42.1	12–32
Bureau of Health staff	9 (three female)	21.43	42–58	45.6	18–36
General practitioners or public health doctors from towns	10 (two female)	23.81	30–55	39.1	7–34
Village doctor group 1	8 (one female)	19.05	26–55	39.7	5–35
Village doctor group 2	7 (all males)	16.67	25–60	38.1	5–40
**Total**	**42**	**100**	**25–60**		**5–40**

The qualitative interviews revealed 31 factors that influenced DRS uptake (figure 1), of which 29 were modifiable and two were unmodifiable. Six of these 31 factors were related to the perspective of service users, whereas the remaining 25 were linked to providers. 11 of the modifiable factors were coded to the DOTS component of government commitment; 12 modifiable factors and one unmodifiable factor were related to case detection, one modifiable factor and one unmodifiable factor were related to standardised treatment, two modifiable factors to drug supply and three were linked to reporting systems.

### Theme 1: Modifiable factors

#### Subtheme 1: Government commitment (11 modifiable factors related)

##### Four factors related to the importance of government funding, consistent policy and effective coordination

The BOH oversees all medical and public health services, including the formulation and adjustment of health policies, financing and monitoring of healthcare deliveries. Coordination between various health institutions is integral to the success of public health initiatives. During group discussions, three main concerns related to the government were raised regarding the quality of screening services and uptake.

First, delays in staff payments negatively affected the quality of primary healthcare services (table 4, quote 1). Second, VDs provided free glucose tests using test strips supplied by the BOH but charged patients when test strips were not provided (table 4, quote 2). This inconsistent charging for the same service led to mistrust among residents, who questioned the reliability of primary healthcare services. Implementing a policy to ensure consistent practices across primary healthcare services could improve public trust and, in return, increase the uptake of DRS at the primary care level. Third, local health authorities did not circulate scheduled screening plans to lower levels (table 4, quote 3). This hindered healthcare providers’ preparation and prevented timely notification of patients. Fourth, many participants and screening team members identified inappropriate preparation as a critical factor explaining the low DRS uptake rate and less effective site coordination (table 4, quote 9).

**Table 4:** List of quotes from diabetic retinopathy screening service suppliers and users

Items	Quotes	Who said
1	The number of PwDM registered at the primary level is unreliable. Village doctors (VDs) were not paid on time, which led to poor quality work at the primary level.	HO
2.	Free glucose tests were given only when free test strips supplied by the local Bureau of Health (BOH) otherwise, we charge 5¥ for each test.	VD
3	Zhenjiang District People’s hospital (ZPH) did not ask the local BOH to announce the screening widely in Shaoguan City.	HO
4	Registration at community health units (CHUs) is not beneficial to patients, but the staff at THUs or community health centers (CHCs), they are paid upon the number of people registered. They do nothing for us but keep our personal medical information if we are registered at the national health system.	PPs.
5	Urban people, and even some rural dwellers, prefer to register at hospitals. Yue Bei Hospital (a tertiary hospital in Shaoguan) had over 30 thousand PwDM registered, and Shaoguan City People’s Hospital (tertiary level) had around 20 thousand PwDM registered. ZPH had only thousands. More people being registered will bring higher DRS uptake.	ZPH leader
6	Yes, we contacted patients directly for DRS and for many other ordinary medical practices without informing VDs before proceeding.	THU staff
7	Patients might have to go to the Department of Medical Insurance for reimbursement of their medical cost, rather to us as many years ago for approval. Thus, the willingness to register is not sufficiently high.	THU staff
8	We need more THU’s engagement in the screening to update patient information and coordinate on-site.	STM
9	As we did not schedule for the screening, many people came in the morning, when we could not handle properly, many of them left due to the long waiting time. This left the afternoon without patients, and we closed the screening early.	STM
10	I went to the screening site at 5 pm on the day, as I was informed, but the screening team left.	PPs
11	No dark rooms were prepared in many THUs. We cannot simply go there for screening without proper preparation.	STM
12	At the DRS pilot stage, we did not arrange any counseling on site as we lacked hands. We should have staff trained in this.	STM and HO
13	We do not talk much with patients about diabetic complications or the DRS, some people suspect being deluded by our medicine sales promotion. We know well PwDM in our own service areas. We did not know there were screening in our places until it started. We would like to be trained to perform better work in health talks.	VDs
14	VDs and health staff at THUs care only about their own records rather than our health. Hospital staff are always too busy to respond to our enquiries.	PPs
15	Rural people regard diabetes mellitus (DM) as a disease only when complications develop.	THU staff
16	A few people still suspect the purpose of the DRS and hesitate to participate. They wondered whether the team would prescribe any medicine to them at DRS. Health information materials and health talks will be helpful in improving the uptake rate.	STM
17	PwDM do not see doctors when without complications. It is people’s own decisions about their health. Some people would take a rest at home or some home-stored medicine as the first action on some diseases. We cannot force them to the examinations.	VDs
18	More health information should be delivered through reliable media, such as TV programs, newspapers, or TikTok.	All
19	Chinese herbal medicine is not helpful to control DM. We do not use it.	PPs
20	Some patients had bad experience with prior community free consultant services. Some even received gifts from the service suppliers. Those who did not receive gifts felt unfairly treated and cheated. Some patients regarded community free activities as unaffordable and unsustainable because these activities are commonly combined with expensive medical items that the supplier wants to promote.	VDs
21	Processes (such as registration, seeing the doctor, examinations, paying the bill, etc) in hospital is complicated and time-consuming when we have to.	PPs
22	Seeing an eye doctor would cost extra consultant fees and cost on fundus image taken (USD18.00 for one eye) in addition to the endocrinologist. We would rather not go for fundus examination with a normal VA. We have not yet been able to obtain the newly established free DRS at hospital.	PPs
23	We do not understand medicine, and would follow up with whatever the doctors say. Cost is really an issue for us as PwDM.	PPs
24	Medicine is available at clinics, hospitals, and pharmaceutical shops and is easy to access.	All
25	Most of PwDM have glucose tests at home.	PPs
26	The eye drops (for pupil dilation) caused my vision to blur for long hours. I was not clearly explained the dilation, and will not go anymore.	PP
27	Some PwDM live over 20 km from the town. It is difficult for them to come for screening in the town.	VDs
28	ZPH was invited to screen in Nanxiong County where we must drive over two hours to reach. It is not convenient for patients to come ZPH, so DRS there was not prepared properly.	ZPH leader

DRS, diabetic retinopathy screening; HO, health officer; PPs, patient participants; PwDM, people with diabetes mellitus; STM, screening team member; THUs, township health units; TV, television; VA, Vision Acuity; VD, village doctor; ZPH, Zhenjiang District People’s Hospital.

##### Three factors related to communication and cooperation among healthcare institutions and professionals

Participants highlighted a lack of coordination between public health staff at THUs and VDs, leading to an ineffective linkage between town and village-level health services. This disconnect was observed and pointed out by the BOH and THU staff (table 4, quote 6). Furthermore, the national chronic disease registration system was poorly integrated with medical insurance and health services, resulting in lower registration rates and reduced DRS uptake (table 4, quote 7). Other healthcare providers have also addressed these concerns. The public health staff from the THUs reported a need for more integration with the hospital system and medical insurance reimbursement process. Enhanced communication and cooperation between town and village health services as well as between township and county-level services were considered essential for optimising primary healthcare delivery (table 4, quote 7). In addition, the staff requested further technical training in diabetes management and chronic disease control. The screening team raised a lack of support from the township health authorities during the DRS (table 4, quote 8). This can be attributed to administrative isolation and systemic shortcomings. These issues can be addressed through frequent interactions and training.

VDs were familiar with all PwDM in their service areas and were knowledgeable about the progression of diabetic complications in these patients, if present. The VDs expressed confidence in their understanding of their patients’ disease states (table 4, quote 13). It is recommended that VDs be recognised as valuable resources for patient management and that their potential administrative role be recognised within public health services, such as DRS.

##### Two factors related to additional training of primary health staff could improve service uptake

The screening team emphasised the need for on-site patient counselling to enhance patients’ understanding of the DRS process. They suggested that well-informed patients could serve as ‘seeds,’ encouraging others to participate in ongoing and future DRS initiatives in the area (table 4, quote 12).

VDs play a crucial role in promoting primary health education, as the first point of contact for health concerns in many communities. However, many VDs lack confidence in delivering health services because of unfamiliarity with health marketing, insufficient training in health promotion and possibly inadequate incentives for conducting such work. Without VDs’ support, improving DRS uptake was difficult (table 4, quote 13).

##### Two factors related to health education

All group discussions highlighted the significant role of DM awareness in influencing health behaviours (table 4, quote 15). Enhancing public knowledge about DM and its potential complications could generate greater demand for DRS.

However, several participants mentioned fraudulent health promotion by pharmaceutical companies, private clinics or unqualified practitioners. These deceptive practices have caused distrust, making people cautious about public health initiatives, even those organised by government institutions, such as the DRS (table 4, quote 16). Consequently, people often hesitate to participate in such programmes.

Many individuals tend to rest or pursue dietary therapy during the early stages of some diseases, instead of seeking medical attention (table 4, quote 17). Participants noted that health information delivered via television, particularly through programmes on Chinese Central Television, was widely trusted. The primary healthcare staff suggested that promoting DRS through television could significantly enhance public awareness and participation (table 4, quote 18). VDs also observed that rural communities had poor health literacy, which was exacerbated by the disorganised health market.

### Subtheme 2: Case detection (12 modifiable factors related)

#### Three factors related to better preparation by service providers could improve screening outcomes

As noted in subtheme 1, a coordinated announcement with a screening schedule, developed through collaboration between THUs and VDs, would allow both healthcare providers and patients to better prepare for screening, potentially leading to increased DRS uptake (table 4, quote 9). In some instances, the screening team left in the late afternoon if no more patients arrived (table 4, quote 10). Additionally, inadequate preparation of the DRS site, such as the absence of a proper dark room and lack of clear patient flow instructions, resulted in poor-quality screening services (table 4, quote 11).

As reported above under the ‘government commitment’ component, factors in this subtheme linked with health education would help improve understanding of the disease and the importance of screening and result in a higher uptake rate.

#### Higher-quality service delivery could improve DRS uptake

All discussion groups mentioned that past negative experiences with healthcare services were barriers to participation in DRS. Some patients mentioned that during previous screenings, fundus images were taken without proper explanation of the results or clarification of the intervals between screenings and referrals. Others expressed confusion about the reports received, indicating a communication gap between healthcare providers and patients (table 4, quotes 13 and 14).

### Subtheme 3: Standardised treatment (1 modifiable factor related)

#### Factors linked with difficulty in accessing medical services

None of the PPs who were regular ZPH visitors reported positive experiences at the hospital. They expressed concerns about unclear instructions from the staff and guides, overcrowding and long waiting times. These challenges have deterred PwDM from visiting the hospital, contributing to low participation in hospital-based DRS (table 4, quote 21).

### Subtheme 4: Uninterrupted drug supply (2 modifiable factors related)

#### Factors related to acceptability and sustainability

Despite the prevalence of unverified marketing for Chinese herbal medicine as a treatment for DM, no PPs have been reported for this purpose (table 4, quote 19). Conflicts have been reported between public health services and private hospitals regarding ‘sales promotion’ activities conducted in local communities (table 4, quote 20). The author believes that initial distrust of DRS will likely diminish over time as high-quality services are delivered and support for the programme grows at the primary healthcare level.

#### Factors related to affordability and accessibility

PPs reported spending a mean of US$70–140 per month on diabetes-related examinations, consultations and medications, which constituted a significant financial burden. This expense was particularly difficult for retired individuals, whose monthly pensions ranged from US$140 to US$422 (table 4, quote 22).

According to the policy, medical insurance reimbursement was available for those with diabetic complications, and individuals without complications were responsible for the out-of-pocket cost of outpatient department (OPD) visits. This financial strain discouraged many people with diabetes from undergoing fundus examinations because of concerns about the additional costs associated with DRS services. Moreover, healthcare professionals did not always address patient concerns regarding these expenses (table 4, quote 23).

Despite cost concerns, the participants noted that they could easily access the medications and glucose testing kits they required through clinics, hospitals and pharmacies (table 4, quote 24). All 15 participants confirmed that they had the necessary equipment and supplies to monitor their glucose levels at home (table 4, quote 25).

### Subtheme 5. Standardised reporting systems

#### Factors related to registration rates and chronic disease management

Healthcare professionals consistently noted that patients registered within national or hospital health systems were more likely to receive effective communication, which led to a higher DRS uptake than that of unregistered individuals (table 4, quote 5). Specifically, urban residents tended to prefer hospital registration, and over time, as patients became regular visitors, communication with staff improved (table 4, quotes 4 and 5). By contrast, rural residents are typically registered within the national chronic disease administration system, facilitating more straightforward access to services without frequent travel (table 4, quote 5).

### Theme 2: Unmodifiable factors

#### Factor 30: Dilation of pupils causing temporary blurred vision (standardised treatment)

One unmodifiable factor that affected patient participation was temporary blurred vision caused by pupil dilation during screening (table 4, quote 26). Pupil dilation was deemed necessary before image capture in this setting because a high rate of ungradable images (over 15%) was encountered without dilation, even with the use of non-cycloplegic cameras. However, a clearer explanation of the dilation process, its effects and the expected recovery time (4–6 hours for normal vision to return) is needed to alleviate these concerns.

Two PPs said that their eyes were uncomfortable for 2 days and their vision was blurred for over 6 hours after dilation. The remaining 13 patients mentioned that the discomfort from dilation lasted only a few hours, with no major concerns expressed regarding the eye drops used during the process (table 4, quote 26).

#### Factor 31: Long distance to screening site (case detection)

Distance was another unmodifiable factor that hindered participation in the DRS. The distance between patients’ homes and the screening site was a significant barrier, as was the distance between the screening location and ZPH, which complicated the hospital’s ability to adequately prepare for and travel to the screening site (table 4, quote 27). Additionally, patients who were identified with issues during screening found it difficult to travel to the ZPH for further examination or treatment, which discouraged follow-up. Hospital leadership also noted this challenge as a factor that reduced the hospital’s motivation to engage in the screening programme (table 4, quote 28).

## Discussion

This study identified 29 modifiable factors affecting DRS uptake and two unmodifiable barriers, pupil dilation and travel distance. By applying the DOTS framework, originally developed for tuberculosis control, we systematically examined the challenges across multiple levels of China’s decentralised healthcare system. By focusing on service delivery processes and provider perspectives, this study provides practical insights into how DRS implementation can be improved, particularly at the township and village levels.

### Health system coordination and governance

Our findings suggest that effective DRS delivery depends heavily on clear scheduling, local ownership and inter-institutional collaboration. A critical barrier to uptake was the inconsistent circulation of screening schedules by the local health authorities, which reduced community preparedness and provider engagement. Delays in staff payments and the ad hoc test strip supply also reflected governance gaps that weakened service credibility and continuity. These challenges align with the broader calls in the literature for embedding eye health into national public health agendas with structured policies, accountability mechanisms and adequate financing.^16^

Strengthening coordination between THUs and VDs has emerged as a priority. VDs hold intimate knowledge of their patients and command local trust; however, they are often excluded from planning and are under-supported in implementation. Recognising their roles as community health educators and referral agents and compensating them accordingly could transform them from passive participants into key actors in the DRS scale-up.^17 18^

To ensure the success of such initiatives, health authorities must be motivated by internal drivers emphasising public health outcomes. Additionally, the financial burden of OPD visits, particularly for elderly patients, must be addressed as these costs are not covered by insurance. Policymakers should consider broader insurance coverage for patients with chronic diseases to alleviate this burden.

### Service delivery and workforce strengthening

Low patient registration at the primary care level further contributes to missed opportunities for screening promotion and follow-up. Improving this would require a combination of government policy, health delivery and long-term development.^19 20^ Both urban and rural health systems must ensure the better integration of chronic disease management systems with eye care services. Investment in training for THU and VD staff, including communication skills and patient education, was widely recommended by the participants to increase trust, uptake and follow-through. The project later reached a 73.4% uptake^14^ after introducing training, clear communication and scheduling, supporting the value of these interventions.

Negative patient experiences,^21^ such as a lack of explanation during screening and confusing referral processes, underscore the need for patient-centred service design. Introducing quality standards, improving screening site readiness (eg, privacy, flow, dark rooms) and training providers to communicate clearly can enhance the acceptability and effectiveness of the service.^22^

### Health promotion, trust and health information systems

Participants consistently noted the importance of health education but also pointed to mistrust caused by misleading information from private actors. This mistrust led to the scepticism of public health initiatives such as DRS. Trusted media, particularly Chinese Central Television, and peer-led promotions have been suggested as promising channels to build awareness and counter misinformation.^23 24^ The findings also highlighted the weak integration between health insurance systems, chronic disease registries and DRS programmes. Better interoperability of health information systems could enable automated reminders, follow-ups and referrals, which are important enablers for screening participation.^25^

### Financial and structural barriers

Although the screening was free, associated costs such as OPD fees for follow-up visits and travel to hospitals were prohibitive, especially for retired individuals with limited pensions. These structural barriers require system-level responses, such as insurance coverage for non-complication visits, transport subsidies or decentralised follow-up systems if the DRS is to be both equitable and scalable.

While pupil dilation and travel distance are largely unmodifiable, clearer patient education about dilation effects and improved transport logistics (eg, mobile follow-up and partnerships with local transport providers) may reduce their practical impact.

While most prior studies^5 26 27^ on DRS uptake have emphasised individual or household-level determinants, our study uniquely focused on modifiable health system-related barriers. Our findings echo reports from Ireland and the Netherlands,^27 28^ where consistent health worker recommendations and provider-patient trust have been shown to increase screening uptake. Notably, fear of DR complications rather than fear of screening was cited by our participants as a motivator for attendance, consistent with findings in Dutch populations.^27^ The knowledge gap and need for training were consistent with other similar studies with telemedicine facilities.^9^

This was a pragmatic service-level study embedded within an ongoing DRS pilot programme in southern China^14^. Following early implementation across Shaoguan, lessons learnt were incorporated into training, outreach and logistics in late 2019, contributing to a significant participation rate of 73.4%,^14^ which continued to rise in subsequent rounds. This reinforces the value of embedded implementation research in adaptive health system reform. Another key strength of the study was the immediate integration of the findings into practice: VDs received training, health talks were held at THUs and promotional materials were distributed.^12 14^ The local BOH had also improved communication by publicly announcing the screening schedule through VDs, media outlets and community leaders.

However, this study had some limitations. It focused primarily on healthcare providers, with limited input from unregistered or rural patients due to the convenience of purposive sampling with only 15 patients registered at the ZPH. The DOTS framework was originally developed for tuberculosis control and therefore primarily reflects the operational components of treatment-oriented public health programmes. While not specifically designed for screening services, it provided a useful systems-level structure for examining governance, case detection processes, resource availability and reporting systems within the DRS programme. However, screening-specific operational factors, such as equipment availability, image quality and patient flow during screening sessions, are not explicitly represented within the original DOTS model. In this study, such issues were incorporated within the broader domains of case detection and service delivery. Future implementation research on screening programmes may benefit from adapting or expanding the framework to explicitly capture these operational aspects. The PPs were from a single hospital, which may limit the representativeness of the sample. Future research should engage more diverse PwDM through in-depth interviews to explore community-level and behavioural barriers, particularly in areas where systemic barriers have already been addressed.

## Conclusion

Improving DRS uptake requires more than offering free services. It requires systemic coordination, consistent communication, patient trust and frontline staff support. Strengthening public health service delivery at the primary level through reliable scheduling, training and integration of VDs, alongside targeted health promotion and insurance reform, is essential for sustainable DRS scale-up. Future work should prioritise patient-centred research and explore scalable innovations to embed DRS into routine chronic disease management across China.

## Data Availability

All data relevant to the study are included in the article or uploaded as supplementary information.
