# Supplementary material for: Contextual factors that affect diabetic retinopathy screening uptake at township health units in southern China: a qualitative study with service providers and users
Source: BMJ Open. 2026 May 12;16(5):e109139. doi: 10.1136/bmjopen-2025-109139 (PMC13182493; doi:10.1136/bmjopen-2025-109139)
Supplement: Supplementary file 1 [file bmjopen-2025-109139.reviewer_comments.pdf]

PEER REVIEW HISTORY

BMJ Open publishes all reviews undertaken for accepted manuscripts. Reviewers are asked to complete a checklist review form and are provided with free text boxes to elaborate on their assessment. These free text comments are reproduced below.

ARTICLE DETAILS

Title (Provisional)

Contextual factors that affect diabetic retinopathy screening uptake at township health units in Southern China: A qualitative study with service providers and users

Authors

Xiao, Baixiang; Chan, Ving Fai; Price-Sanchez, Carlos; Piyasena, Mapa Prabhath; Wang, Yanfang; Wan, Qiong; Congdon, Nathan

---

VERSION 1 - REVIEW

**Reviewer**	**1**
**Name**	**Ramchandran, Rajeev**
**Affiliation**	**University of Rochester, Ophthalmology**
**Date**	**13-Oct-2025**
**COI**	**None**

Very important work.

Needs to define eye exam for patients with diabetes mellitus - does it mean camera based exam or in person dilated eye exam, or both.

The focus of results is on providers and staff and not patients, methods states patients were interviewed, please make it more clear in methods/results why providers were the focus.

Discussion should be expanded to include strengths and limitations of study and next steps of the research team. Comparisons to published studies should be used. The use of real time improvements in the program addressing DRS could be compared to this being done in other programs in the published literature. Incorporating the literature review on what other qualitative evaluations of DRS - telemedicine and in person exams have said should be done.

**Reviewer**	**2**
**Name**	**Mishra, Chitaranjan**
**Affiliation**	**Trilochan Netralaya, Sambal Pur, Odisha, India**

**Date**	**08-Dec-2025**
**COI**	**None**

This is a good qualitative study focusing on Contextual factors that affect diabetic retinopathy screening uptake at township health units in Southern China.

Here are some comments.

1. All group discussions were arranged within one week of early 2019. But this article was submitted in 2025. Any reason for a long gap in the submission of the manuscript related to the study?

2. Two representative patient groups with n=15 is a very small number. This is to be mentioned in the limitations of the study.

3. How was the selection of the persons in the patient groups and the health care providers done? Was it a random sampling?

4. Similar studies involving the patients' perceptions about the DR screening programs from other parts of the world need to be discussed with references.

5. The discussion on the cameras involved in the DR screening is limited. Please elaborate on this with references.

**Reviewer**	**3**
**Name**	**Chen, Yanyan**
**Affiliation**	**Wenzhou Medical University**
**Date**	**03-Feb-2026**
**COI**	**None**

This qualitative study examines factors influencing the uptake of diabetic retinopathy screening (DRS) at township health units in southern China, using focus group discussions and thematic analysis informed by the DOTS framework adopted from tuberculosis control programs. The research design is sound, data collection is systematic, and the analytical process is transparent, meeting fundamental methodological standards for qualitative inquiry. The study identifies 31 influencing factors (29 modifiable and 2 non-modifiable) and offers practical recommendations for improving screening participation.

Comments：

1. Sampling and representativeness

The study relies primarily on registered patients from a single hospital, which may limit the representativeness of the sample. It is recommended to emphasize this limitation more clearly in the “Limitations” section and to suggest that future studies expand the sample to include unregistered patients, particularly through in-depth interviews, to better capture diverse perspectives.

2. Data collection process

The focus groups were facilitated by the project lead, whose known role may have introduced social desirability bias, especially among healthcare providers. To mitigate this, the “Methods” section should briefly describe how neutrality was maintained—for example, through the use of open-ended questions, encouragement of contrasting views, and reflexive practices.

3.Development of interview questions

The manuscript does not describe the rationale or process for formulating the two core interview questions. It is recommended to clarify in the "Methods" section how these questions were developed—for instance, based on literature review or expert consultation.

4. Data analysis and thematic derivation

Although the DOTS framework was applied, the process of categorizing the 31 factors into the five predefined themes is not fully explained, and some overlap between themes (e.g., health education and case detection) is evident. It is suggested to include a summary table in the “Results” linking each factor to the relevant DOTS component, thereby enhancing the transparency of the coding process.

5. Applicability of the theoretical framework

The DOTS framework was originally designed for tuberculosis control and may not fully encompass factors specific to screening-based services such as DRS (e.g., equipment availability, image quality). A brief discussion in the “Discussion” section on the suitability and limitations of the DOTS framework in this context—including whether any screening-specific factors were overlooked—would strengthen the methodological reflection.

6. Description of data saturation

The manuscript states that data collection continued “until no more factors could be identified” but it does not specify how saturation was determined (e.g., through coding consistency, team deliberation, or iterative review). It is recommended to supplement the “Methods” section with a clear description of the criteria and process used to assess data saturation, supported by relevant methodological references where appropriate.

VERSION 1 - AUTHOR RESPONSE

Reviewer: 1
Dr. Rajeev Ramchandran, University of Rochester

Comments to the Author:
Very important work.

Needs to define eye exam for patients with diabetes mellitus - does it mean camera based exam or in person dilated eye exam, or both.
Response: This means both. It is now referred and articulated.

The focus of results is on providers and staff and not patients, methods states patients were interviewed, please make it more clear in methods/results why providers were the focus.
Response: Thanks for this good point, which is now added to the methods.

Discussion should be expanded to include strengths and limitations of study and next steps of the research team. Comparisons to published studies should be used. The use of real time improvements in the program addressing DRS could be compared to this being done in other programs in the published literature. Incorporating the literature review on what other qualitative evaluations of DRS - telemedicine and in person exams have said should be done.
Response. Many thanks for the good point. Studies related to telemedicine are now added to the methods and discussions.

Reviewer: 2
Dr. Chitaranjan Mishra, Trilochan Netralaya, Sambal Pur, Odisha, India
Comments to the Author:
This is a good qualitative study focusing on Contextual factors that affect diabetic retinopathy screening uptake at township health units in Southern China.

Response. Thanks. Agree.
Here are some comments.

1. All group discussions were arranged within one week of early 2019. But this article was submitted in 2025. Any reason for a long gap in the submission of the manuscript related to the study?
Response. Thanks for this question. We focused on the main result of the prevalence and waited for it published before went on working on the qualitative study report.

2. Two representative patient groups with n=15 is a very small number. This is to be mentioned in the limitations of the study.

Response. Thanks. Added now.
3. How was the selection of the persons in the patient groups and the health care providers done? Was it a random sampling?
Response. Thanks for the question. As mentioned at methods. These were purposively selected.

4. Similar studies involving the patients' perceptions about the DR screening programs from other parts of the world need to be discussed with references.
Response. Thanks. Done.
5. The discussion on the cameras involved in the DR screening is limited. Please elaborate on this with references.
Response. Thanks. Done.

Reviewer: 3
Dr. Yanyan Chen, Wenzhou Medical University
Comments to the Author:
This qualitative study examines factors influencing the uptake of diabetic retinopathy screening (DRS) at township health units in southern China, using focus group discussions and thematic analysis informed by the DOTS framework adopted from tuberculosis control programs. The research design is sound, data collection is systematic, and the analytical process is transparent, meeting fundamental methodological standards for qualitative inquiry. The study identifies 31

influencing factors (29 modifiable and 2 non-modifiable) and offers practical recommendations for improving screening participation.

Comments：

1. Sampling and representativeness

The study relies primarily on registered patients from a single hospital, which may limit the representativeness of the sample. It is recommended to emphasize this limitation more clearly in the “Limitations” section and to suggest that future studies expand the sample to include unregistered patients, particularly through in-depth interviews, to better capture diverse perspectives.

Response. Yes as discussed and some more points added.

2. Data collection process

The focus groups were facilitated by the project lead, whose known role may have introduced social desirability bias, especially among healthcare providers. To mitigate this, the “Methods” section should briefly describe how neutrality was maintained—for example, through the use of open-ended questions, encouragement of contrasting views, and reflexive practices.

Response. Many thanks for the good point. These are now added.

3.Development of interview questions

The manuscript does not describe the rationale or process for formulating the two core interview questions. It is recommended to clarify in the "Methods" section how these questions were developed—for instance, based on literature review or expert consultation.

Response. Many thanks for your reminder. As the purpose of the study was for the improvement of the program, so that questions were developed from the program by the program staff together with local health staff. These are now added.

4. Data analysis and thematic derivation

Although the DOTS framework was applied, the process of categorizing the 31 factors into the five predefined themes is not fully explained, and some overlap between themes (e.g., health education and case detection) is evident. It is suggested to include a summary table in the “Results” linking each factor to the relevant DOTS component, thereby enhancing the transparency of the coding process.

Response: Thank you for this helpful suggestion. To improve transparency in the analytical process, we have clarified in the Methods section how the identified factors were mapped to the five components of the DOTS framework using a deductive thematic approach. During coding, each factor derived from the focus group discussions was reviewed and assigned to the DOTS component that most closely reflected the service delivery domain in which the issue occurred. Where overlap occurred (for example, health education influencing case detection), the factor was categorised according to its primary operational function within the screening programme. Figure 1 shows the identified factors to the relevant DOTS components.

5. Applicability of the theoretical framework

The DOTS framework was originally designed for tuberculosis control and may not fully encompass factors specific to screening-based services such as DRS (e.g., equipment availability, image quality). A brief discussion in the “Discussion” section on the suitability and limitations of the DOTS framework in this context—including whether any screening-specific factors were overlooked—would strengthen the methodological reflection.

Response: Thank you for highlighting this important methodological point. We agree that the DOTS framework was originally designed for tuberculosis control and may not fully capture all dimensions relevant to screening-based services such as diabetic retinopathy screening. We have added a paragraph in the Discussion section reflecting on the suitability and limitations of applying the DOTS framework to DRS programmes, noting both its usefulness for analysing health system structures and the potential limitations in capturing screening-specific operational factors.

6. Description of data saturation
The manuscript states that data collection continued “until no more factors could be identified” but it does not specify how saturation was determined (e.g., through coding consistency, team deliberation, or iterative review). It is recommended to supplement the “Methods” section with a clear description of the criteria and process used to assess data saturation, supported by relevant methodological references where appropriate.

Response: Thank you for this important suggestion. We have clarified the process used to determine data saturation in the Methods section. Saturation was assessed through iterative review of transcripts and coding consistency during analysis. When no new factors influencing DRS uptake emerged from subsequent group discussions and existing themes were repeatedly confirmed, the research team agreed that thematic saturation had been reached.

Reviewer: 1
If you have selected ‘Yes’ above, please provide details of any competing interests.: no applicable

Reviewer: 2
If you have selected ‘Yes’ above, please provide details of any competing interests.: Not Applicable

Reviewer: 3
If you have selected ‘Yes’ above, please provide details of any competing interests.: Not applicable.

VERSION 2 - REVIEW

**Reviewer**	**1**
**Name**	**Ramchandran, Rajeev**
**Affiliation**	**University of Rochester, Ophthalmology**
**Date**	**28-Mar-2026**
**COI**	

All comments have been adequately answered
